# Biological Monitoring of Air Pollutants and Its Influence on Human Beings

**DOI:** 10.2174/1874120701509010219

**Published:** 2015-08-31

**Authors:** Shihong Cen

**Affiliations:** Zhengzhou University of Industrial Technology, China

**Keywords:** Air pollutants, biological monitoring, damage symptoms, health, plants

## Abstract

Monitoring air pollutants via plants is an economic, convenient and credible method compared with the traditional ways. Plants show different damage symptoms to different air pollutants, which can be used to determine the species of air pollutants. Besides, pollutants mass concentration scope can be estimated by the damage extent of plants and the span of polluted time. Based on the domestic and foreign research, this paper discusses the principles, mechanism, advantages and disadvantages of plant-monitoring, and exemplifies plenty of such plants and the minimum mass concentration and pollution time of the plants showing damage symptoms. Finally, this paper introduced the human health effects of air pollutants on immune function of the body, such as decrease of the body's immune function, decline of lung function, respiratory and circulatory system changes, inducing and promoting human allergic diseases, respiratory diseases and other diseases.

## 1. INTRODUCTION

In recent years, along with the quantity and traffic congestion coefficient increase, industrial waste gas emissions of road vehicles, a number of large and medium-sized city air quality gradually decline, coupled with the frequent occurrence of fog and haze, air pollution is becoming more and more serious [1]. Currently, people can’t accomplish at one stroke to improve air quality. Increasing the investment in improving the air quality at the same time, people can only follow the prescribed order to conduct rigorous testing of atmospheric pollutants, in order to understand and master the air quality and related issues of people's contact at any time.

## 2. CONCEPT AND BASIC CONTENT OF AIR POLLUTION AND ENVIRONMENTAL MONITORING

### 2.1 Air pollution, Air Pollutants and Total Quantity Control of the Pollutant Exposition

Air pollution, as the name implies, refers to the number of harmful substances in the atmosphere of concentration and residence time more than the allowed range of atmospheric environment, which is beyond the capability of diffusion and dilution, the air, the air quality deterioration, brought bad influence directly or indirectly to human health and ecological environment; atmospheric pollutant is produced by natural process refers to the human activities and the pollution to the atmospheric environment, this paper mainly refers to the pollutants produced by human activities including industrial and agricultural production, daily life and the means of transport, the total amount of pollutants; control refers to a region, the total emission of air pollutants discharged into the region closely monitoring and control in a certain period of time, so that the air dilution and diffusion, and atmospheric environment pollution [2].

### 2.2 Air Pollution Sources and Some Common Air Pollutants

Some common air pollutants are SO_2_, suspended particles mainly PM_2.5_, NO_X_, CO, VOCS (Volatile Organic Compounds), heavy metal, radioactive substances, photochemical oxide, etc. The cause of these air pollutants are the major factors of the discharge of pollutants exceed the atmospheric saturation, pollutants, pollution emission height distance, air self-purification ability. Table **1** shows pollutants in different time over different concentration, which will lead to air pollution.

### 2.3 Environmental Monitoring

Environmental monitoring means intermittent detection for air pollutants, pollution sources and other content by department of environmental monitoring, to forecast the environmental problems and to prevent environmental problems.

## 3. BIOLOGICAL MONITORING OF AIR POLLUTANTS

Through the continuous observation and study, people found that some plants show some reaction for harmful gas, which can be used as a biological indicator of certain plants to monitor atmosphere. Plants under different air pollutants harm will exhibit different symptoms. These symptoms can be used to judge the type of atmospheric pollutants, and to estimate its concentration range according to the damage extent of plants and the time of pollution [3]. Compared with traditional monitoring method, using plants to monitor air pollutants is an economic, simple and reliable method.

### 3.1 The Principle of Biological Monitoring

#### 3.1.1 The Mechanism of Monitoring Air Pollutants by Plants

The basic principle of monitoring air pollutants by plants is using the biological effect of them for air pollutants. That's to say, the damage symptoms of plants, mainly leaves, is related with the types, concentration and contacting time of pollutants. Air pollution situation, especially the types of air pollutants, is evaluated by the community types of injured plants and symptoms of victims. Moreover, the concentration of pollutants is judged by the victimization levels and contamination time of plants.

Atmospheric pollutants come in plants through leaf stomata. Leaf is an important part of plant, and is the main organ for photosynthesis and transpiration. Air pollutants can directly damage plant leaves. The victimization levels are directly related with that whether air pollutants coming into stomata or not and how much. It’s monitored from symptoms of plants that which kind and how much of concentration of harmful gas [4].

#### 3.1.2 Symptoms of Endangered Plants by Several Kinds of Harmful Air Pollutants

The damage symptoms on plants of several main pollutants in the atmosphere are shown in Table **2**.

#### 3.1.3 Monitoring of Concentration Plant

Plants can be used to monitor SO_2_ are alfalfa, sesame, moss, spinach, carrot, sweet potato, cucumber, oat, soybean, cotton, tobacco, pepper, *Zinnia*, wheat, red sage, rose, chrysanthemum, carnation, apple tree, Chinese rose, silk tree, plum, sycamore, poplar, birch, pine, cedar, larch, etc. It was monitored that when the atmosphere SO_2_ concentration was 3.4 mg/m^3^, symptoms of alfalfa and sesame were visible after exposed for one hour; when the atmosphere SO_2_ concentration was 0.14 ~ 1.4 mg/m^3^, symptoms of spinach, cucumber and oats were visible after exposed for 8 hours, or 3 ~ 11mg/m^3^ for 30 minutes.

*Gladiolus* is the most commonly used plant for monitoring fluoride [5]. It will be damaged in 9 * 10^-4^ mg/m^3^ HF for 2 ~ 3 h or 9 * 10^-3^ mg/m^3^ for 20 min. In addition, apricot, plum, plum, grape, tulip, garlic, jade hairpin, moss, tobacco, buckwheat, corn, tomato, mango, peach, peach tree, elm, cedar and other plants can also be used for monitoring of fluoride.

O_3_ can be monitored by tobacco, tomato, pea, spinach, potato, oat, crab grass, peanut, lilac, peony, radish, muskmelon and American pine. When the mass concentration of O_3_ in the atmosphere reaches 0.1 * 10^-3^ to 0.26 * 10^-3^ mg/m^3^ for 2 ~ 4 h, these plants will show symptoms.

PAN can be monitored by *Petunia*, chickweed, bluegrass, lettuce, bean, tomato, tobacco, mustard, etc. When the mass concentration of PAN in the atmosphere is 0.4 ~ 0.5 mg/m^3^, there will be visible symptoms after 4h.

NO_2_ can be monitored by tobacco, oat, wheat, corn, carrots, tomato, potato, onion, bean, *Citrus*, melon and so on. Under weak light, these plants will show symptoms under 5 ~ 6 mg/m^3^ NO_2_ for 2 ~ 3 h [6].

Chlorine and chloride can be monitored by *Acer negundo*, larch, *Pinus tabulaeformis*, kapok, fake forsythia, apple, peach, alfalfa, barley, buckwheat, corn, cabbage, spinach, radish, leek, onion, onion, tomato, melon, beans, sunflower, etc. These plants will show symptoms when the content of Cl_2_ in the air reaches 0.15 ~ 3 mg/m^3^ and lasts for 3 ~ 5 h.

### 3.2 Monitoring Methods

#### 3.2.1 Determining Atmospheric Pollution According to the Visible Symptoms

(1) Monitoring air pollution using indicator plants to guard. For instance, planting a variety of sensitivity of different plants around the plant, not only beautify the environment but also monitoring the pollution of the environment.

(2) Estimating the level of air pollution by plant communities. Because of the different sensitivity to pollution, the reaction of different kind of plants in the community is significantly different for air pollution. Table **3** shows the survey results about the plant community around chemical factories which had SO_2_ emission [7].

#### 3.2.2 Using Lichens to Monitor Air Pollution

Lichens are symbiotic bacteria and algae, most can endure the harsh environmental conditions. But lichens are sensitive to SO_2_, H_2_S and other air pollutants, and even a small amount of toxic substances can affect their growth, and induce their death [8].

We can investigate lichen species, number and distribution area around the contaminated area to estimate the pollution. Table **4** is a survey for genus, species and number of lichens at different distances from a factory [9].

#### 3.2.3 Using Moss to Monitor Air Pollution 

Moss is a sensitive indicator of plant just after the lichen. When exposed to air pollution, the moss will appear black or brown phenomenon which is obvious. Moss communities will decline seriously and the species diversity of moss species will continue to decrease until disappear in long-term pollution. Real *et al.* first used moss to monitor fluoride concentration in the environment [10].

#### 3.2.4 Estimating Pollution According to the Content of Toxicant In Plant Leaves

Leaf is the main organ to absorb atmospheric pollutants. Therefore, determination of leaf content of pollutants can explain the contamination in the area [11].

#### 3.2.5 Analysis by Tree Ring

The tree ring can reflect the pollution history of several years, average tree ring contaminated narrowed. Also the ring material was determined by X-ray, the pollution situation, the year the proportion of serious pollution of the small wood.

#### 3.2.6 Other Monitoring Methods

The changes of photosynthesis and other physiological indexes of plants can reflect the pollution of the atmospheric environment, such as the determination of plant photosynthesis to produce oxygen capacity and content of chlorophyll *a*.

## 4. EFFECTS OF AIR POLLUTANTS ON HUMAN HEALTH

Human health hazards of air pollution. Under low concentration and long-term air pollution, body's immune and lung function will decline, and respiratory and circulatory system will change, inducing and promoting human allergic diseases, respiratory diseases and other diseases [12, 13].

Inhalable particulate matter PM_10_ and fine particulate matter PM_2.5_ is most harmful for health. They can come into bronchial, lung and deposition by breathe, long-term effect is the direct cause of bronchial inflammation, especially chronic obstructive pulmonary disease; they can come even through the alveolar and into the blood, which is one of the major risk factors for cardiovascular disease and pulmonary heart disease. These fine particles are also the carrier of toxic and harmful ingredients, such as bacteria, virus, heavy metals and organic compounds, which are carcinogenic and will promote cancer [14-16].

SO_2_ is oxidized to acid mist in the air, which can produce acute stimulation on the human eye conjunctiva, nose and respiratory tract mucosa, and cause increased bronchoconstriction, airway resistance. Acid mist may cause lung inflammation and pulmonary edema in lung tissue. Patients whose pulmonary are insufficient, the elderly and children are particularly sensitive to the stimulation of SO2. It’s harmful for chronic bronchitis, emphysema and chronic lower respiratory diseases such as chronic obstructive pulmonary disease (COPD) if in long term effects [17].

NO_x_ has low water solubility, and can invade the deep respiratory bronchioles and alveoli. After long-term inhalation, NO_x_ will be oxidized by active substance oxidation surfactant, which produces nitrite and nitrate, and induces lung tissue corrosion and stimulation, causing bronchiolitis obliterans and pulmonary edema. NO_x_ is an important substance is the formation of photochemical smog. Smog which has strong oxidation can cause symptoms of respiratory system and cardiovascular system, such as eye irritation, difficulty breathing, headache, chest tightness and shortness of breath, which is more pronounced for some people who have heart disease and lung disease [17, 18].

## CONCLUSION

Atmospheric pollution is closely related to people's life. Although we can using the biological method of monitoring air pollutants to take measures to prevent the disease, the most fundamental way for our health is that do everything you to reduce emissions of pollutants. Only in this way, environment will become cleaner, and our children will thrive under the blue sky and white clouds.

## Figures and Tables

**Table 1.** The average concentration of air pollutants at different time.

Air pollutants	Mean time	Primary concentration limit on average /μg·m-3	Secondary concentration limit on average /μg·m-3
SO2	1h	150	500
	24h	50	150
	1a	20	60
NO2	1h	200	200
	24h	80	80
	1a	40	40
CO	1h	10	10
	24h	4	4
O3	1h	160	200
	8h	100	160
PM10	24h	50	150
	1a	40	70
PM2.5	24h	35	75
	1a	15	35

**Table 2.** Symptoms of endangered plants by several kinds of harmful air pollutants.

Air pollutants	Damage mechanism	Injury spot area	Injury spot shape	Injury spot color	Age and degree of the damaged leaf
SO2	Induce plasmolysis of spongy cells and palisade cells, then shrink or collapse, chlorophyll decomposition	Mainly pulse, occasionally leaf margin	Irregular points, block, clear boundaries	Brown, red brown	Expanded leaves > old leaves and mature leaves > unfolded leaves
fluoride	Induce plasmolysis of mesophyll and cell	Mainly leaves and margin, occasionally pulse	A strip or band	Pale brown	Young leaves > mature leaf > old leaf
O3	Destruct cell wall of palisade tissue and epidermal cells, oxidize glucose	Mainly leaf surface, occasionally pulse	Scattered dense punctate	Brown, tawny	Mature leaves > young leaves > old leaves
Peroxyacyl nitrates(PAN)	Induce leaves to shrink, loss water, and then be filled into the air	Mainly blade back, occasionally leaf tip	Glass, necrotic zone	Silvery white, brown, tan	Young leaves tip and old leaves base vulnerable
NO2	Break cell	Pulse	Irregular spot or whole leaf spot	White, tawny, brown	Young leaves vulnerable
Chlorine and chloride	Destruct chlorophyll	Pulse	Point block boundaries or transition	Severe chlorosis, bleaching	Mature leaves vulnerable

**Table 3.** Plants situation around 30 ~ 50 m of chemical factories.

Plant	Situation
Hung ling wood, poplar, Canada, Sabina chinensis, towel gourd	Above 80% leaves suffered and even fell off, leaves had obvious large scars, part of the plants died.
Sunflower, onion, corn, chrysanthemum, morning glory	About 50% foliar surface was damaged, there were point and block-shaped scars between leaf veins
Chinese rose, rose, Chinese wolfberry, cedar, cypress	About 30% foliar surface was damaged, there were a little few point and block-shaped scars between leaf veins
Grape, honeysuckle, medlar, purslane	About 10% foliar surface was damaged, there were a little few point -shaped scars between leaf veins
Magnolia, big leaf boxwood, gardenia, wintersweet	No obvious symptoms

**Table 4.** An investigation for genus, species and number of lichens at different distances from a factory.

Sampling number	Distance from the factory /m	Number of genus	Number of species
A	100	3	5
B	450	3	7
C	1100	4	9
D	1350	5	11
E	1600	7	15
F	2600	9	26
G	6500	12	39
